# No-Reference Video Quality Assessment Using Multi-Pooled, Saliency Weighted Deep Features and Decision Fusion

**DOI:** 10.3390/s22062209

**Published:** 2022-03-12

**Authors:** Domonkos Varga

**Affiliations:** Ronin Institute, Montclair, NJ 07043, USA; domonkos.varga@ronininstitute.org

**Keywords:** no-reference video quality assessment, convolutional neural network, decision fusion

## Abstract

With the constantly growing popularity of video-based services and applications, no-reference video quality assessment (NR-VQA) has become a very hot research topic. Over the years, many different approaches have been introduced in the literature to evaluate the perceptual quality of digital videos. Due to the advent of large benchmark video quality assessment databases, deep learning has attracted a significant amount of attention in this field in recent years. This paper presents a novel, innovative deep learning-based approach for NR-VQA that relies on a set of in parallel pre-trained convolutional neural networks (CNN) to characterize versatitely the potential image and video distortions. Specifically, temporally pooled and saliency weighted video-level deep features are extracted with the help of a set of pre-trained CNNs and mapped onto perceptual quality scores independently from each other. Finally, the quality scores coming from the different regressors are fused together to obtain the perceptual quality of a given video sequence. Extensive experiments demonstrate that the proposed method sets a new state-of-the-art on two large benchmark video quality assessment databases with authentic distortions. Moreover, the presented results underline that the decision fusion of multiple deep architectures can significantly benefit NR-VQA.

## 1. Introduction

Measuring the quality of digital videos has been a hot and important research topic in the literature. Namely, digital videos undergo a series of processes, i.e., compression or transmission, before they are displayed [1]. Moreover, each process affects the video in a certain way, and in most cases it will introduce some type of artifact or noise. These artifacts, which can be blur, geometric distortions, or blockiness artifacts from compression standards, degrade the perceptual quality of the digital video. In the literature, video quality assessment (VQA) is divided into two broad classes: subjective and objective. Specifically, subjective VQA deals with collecting quality ratings from a group of human beings using a set of videos. The experiments can be carried out either in a laboratory environment [2] or a crowd-sourcing process [3] via online. The quality ratings, which were obtained from human observers, are averaged into one number—the mean opinion score (MOS)—to characterize the perceptual quality of each considered video sequence. In addition, subjective VQA deals with many aspects of video quality measurement, such as the selection of test video sequences, grading scale, time interval of video presentation to human subjects, viewing conditions, and selection of human participants [4,5]. As a result, subjective VQA provides benchmark databases [5,6,7] which contain video sequences with their corresponding MOS values. These databases are extensively applied as training or testing data by different objective VQA methods which aim to construct mathematical models for accurately estimating the perceptual quality of video sequences.

Objective VQA can be classified with respect to different factors. The most common way of classification in the literature [8,9,10,11] is based on the availability of the pristine, reference videos, whose visual quality is considered perfect for the objective VQA algorithm. Specifically, objective VQA is categorized into three groups: full-reference (FR), reduced-reference (RR), and no-reference (NR) ones. For FR-VQA algorithms, the entire reference video is available, while NR-VQA algorithms have no access to the reference videos. On the other hand, some representative features of the reference video are available for an RR-VQA algorithm. In the literature, the construction of an NR-VQA algorithm is considered the most challenging [12,13] due to the complete lack of information about the reference videos and the most useful, as reference videos are not available in many practical, everyday applications, such as video streaming [14].

Recently, the deep learning paradigm has dominated the field of computer vision, image, and video processing [15,16,17,18,19]. Moreover, the field of NR-VQA was also heavily influenced by this trend [20,21,22,23,24,25]. The present paper’s specific contributions are a novel, innovative deep learning based approach for NR-VQA that relies on a set of in parallel pre-trained convolutional neural networks (CNN) to characterize versatitely the potential image and video distortions. More specifically, temporally pooled and saliency weighted video-level deep feature vectors are compiled from a set of pre-trained CNNs and mapped onto perceptual quality scores independently from each other using trained regressors. Finally, the quality scores coming from the different regressors are fused together to get the perceptual quality of the input video sequence. We empirically corroborate that the decision fusion of multiple deep architectures is able to significantly improve the performance of NR-VQA. Namely, extensive experiments were carried out on two large benchmark VQA databases (KoNViD-1k [7] and LIVE VQC [26]) with authentic distortions.

The remainder of this paper is structured as follows. In Section 2, we introduce the status of research in NR-VQA. In Section 3, we describe the overall architecture of the proposed method. In Section 4, we describe the applied benchmark databases that were used to train and test the proposed architecture. Moreover, the applied evaluation metrics and environment are also described. In Section 5, we introduce experiments designed to evaluate performance of the method and describe the experimental results. In Section 6, we give the conclusion and clarify the next work.

## 2. Literature Review

Due to the complexity of the human visual system (HVS), NR-VQA is a very challenging task. Therefore, a huge amount of studies and papers can be found in the literature dealing with NR-VQA. Methods found in the literature can be classified into three large groups: bitstream-based, pixel-based, and hybrid models. Specifically, bitstream-based methods analyze the video frame headers and the decoded packets to estimate digital videos’ perceptual quality. A typical example of this group is the QANV-PA (Quality Assessment for Network Video via Primary Analysis) method [27]. Namely, the authors extracted first five video frame level parameters, i.e., quantization parameter, frame display duration, number of lost packets, frame type, and bitrate. Moreover, a pooling procedure of the frame-level parameters was also introduced to characterize perceptual video quality. In contrast, Lin et al. [28] built their model on three factors, i.e., quantization parameter, bit location, and motion. Yamagishi and Hayashi [29] used a packet-layer model for estimating the perceptual quality of internet protocol television (IPTV) videos. Specifically, the authors analyzed the packet-headers of videos and extracted quality-aware features, such as bit rate and packet-loss frequency. Bitstream-based methods perform well in network video applications, such as video conferencing or IPTV, but cannot be exploited for general applications [30].

Pixel-based NR-VQA methods take the raw video signal as input for quality prediction. Different natural scene statistics (NSS) approaches are very popular in the literature [31,32,33]. The main idea behind NSS is that natural images and videos possess certain statistical regularities that are corrupted in the presence of noise. The discrete cosine transform (DCT) [34] domain is very popular to quantify the deviation from “natural” statistics in the literature. For instance, Brandao and and Queluz [35] used DCT coefficients to fit different probability density functions (PDF) on them. Specifically, the parameters of these PDFs were estimated by maximum likelihood and were applied for local error estimation. This was followed by a perceptual spatio-temporal weighting model to quantify overall perceptual quality. In contrast, Saad et al. [36] first took the difference of consecutive video frames and applied on these difference images local block-based DCT. Next, the DCT coefficients were modeled by a generalized Gaussian distribution (GGD) and the parameters of the GGD were considered as quality-aware features. Moreover, these quality-aware features were combined with motion coherency vectors and mapped onto quality scores with the help of support vector regressor (SVR). In contrast, Li et al. [37] utilized 3D-DCT for feature extraction instead of frame level features but similarly to [36] the feature vectors were mapped onto quality scores with an SVR. Similarly to the work in [37], Cemiloglu and Yilmaz [38] utilized 3D-DCT for feature extraction but first the video content was segmented into cubes of various sizes relying on spatial and motion activity measurement. In contrast, Zhu et al. [39] extracted video frame level features from each video frame. Specifically, six feature maps were generated for every video frames using DCT. Subsequently, five quality-aware features were extracted from the feature maps and temporally pooled together to form video-level feature vectors which were mapped onto quality scores with a neural network. In [40], the authors improved further this method by introducing new frame level features. Besides DCT, other transform domains are also popular in the literature, such as shearlet [41], wavelet [42], or complex wavelet [43] transform domains. Another line of works extracted different optical flow statistics to compile quality-aware feature vectors. For example, Manasa et al. [44] characterized the inconsistencies in the optical flow both at image patch and video frame level. Specifically, intra-patch and inter-patch level irregularities were measured and combined with the correlation between successive frames. At the frame level, the magnitude difference between two consecutive frames in the optical flow was measured. Similarly to the previously mentioned methods, the extracted features were mapped onto quality scores with a trained SVR. In contrast, Men et al. [45] combined spatial features, such as contrast or colorfulness, with temporal features derived from optical flow to compile feature vectors.

Recently, deep learning techniques have become very popular in pixel-based algorithms. Moreover, deep learning has also gained significant attention in the related fields, such as stereoscopic [46] and omnidirectional [47] image quality assessment, image superresolution [48], or stereoscopic VQA [49]. For instance, Li et al. [41] trained a CNN (convolutional neural network) from scratch on 3D shearlet transform coefficients extracted from video blocks for perceptual video quality estimation. In contrast, Ahn and Lee [20] fused hand-crafted and deep features to compile quality-aware feature vectors for video frames. Next, a frame to video feature aggregation procedure was applied and the resulting vector was regressed onto quality scores. Agarla et al. [50] applied deep features extracted from pretrained CNNs for predicting image quality attributes, such as sharpness, graininess, lightness, and color saturation. Based on these quality attributes, frame-level quality scores were generated and used for perceptual video quality estimation using a recurrent neural network. In [51], the authors improved further the previously mentioned method by introducing a sampling algorithm that eliminates temporal redundancy in video sequences by choosing representative video frames.

Hybrid methods combine the principles of bitstream-based and pixel-based algorithms. For instance, Konuk et al. [52] combined a spatiotemporal feature vector with average bit rate and packet loss ratio. In [53], the authors predict the perceptual quality of videos transferred over the universal mobile telecommunication system by combining sender bitrate, block error rate, and mean burst length in a nonlinear regression analysis. Similarly, Tao et al. [54] investigated video quality over IP networks.

For comprehensive surveys about NR-VQA, we refer readers to the works in [55,56,57],

## 3. Proposed Method

**Figure 1:**
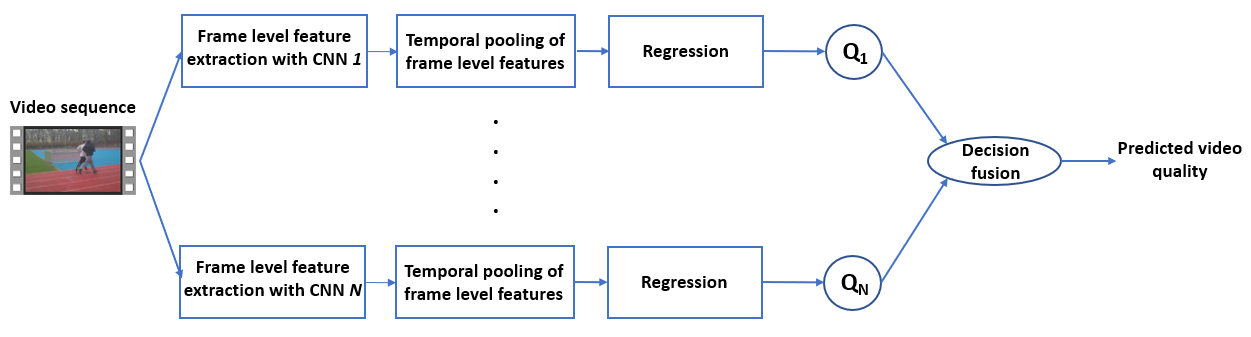
High-level workflow of the proposed algorithm. Temporally pooled and visual saliency weighted deep features are extracted from each video sequence with the help of multiple pre-trained CNNs independently from each other. Next, the extracted deep feature vectors are mapped onto perceptual quality scores. These scores are fused together to obtain the estimated perceptual quality of the input video sequence.

The high-level workflow of the proposed NR-VQA algorithm is depicted in Figure 1. As it can be seen from this figure, multiple temporally pooled video-level feature vectors are compiled with the help of deep frame-level feature vectors extracted from each video frame using a diverse set of pre-trained CNNs. Next, these video-level feature vectors are mapped onto perceptual quality scores independently from each other. Finally, these scores are fused together to obtain an estimation for the perceptual quality of the input video sequence.

The main properties of the applied pre-trained CNNs are summarized in Table 1. Specifically, it can be seen that seven different architectures were utilized from which six ones were trained on ImageNet [58] and one CNN was trained on Places-365 [59] dataset. The main idea behind this layout is that deep features with multiple sources could better capture possible image distortions than a single one [60]. Namely, the computer vision research community has pointed out that internal activations of pre-trained CNNs as deep features are able to provide powerful representations [61,62,63]. Moreover, CNNs can capture spatial and temporal dependencies in an image with the help of relevant convolutional filters [64]. Further, the first layers of a CNN capture low-level image features, i.e., edges, colors, or blobs, while the network also captures high-level features which are important in understanding of image semantics [65,66]. The previously mentioned dependencies and features are obviously degraded in the presence of image noise and distortion. Therefore, they can be utilized as quality-aware features.

As already mentioned, the temporally pooled frame-level features are mapped onto perceptual quality scores using a regression machine learning technique. In this paper, we show experimental results with the usage of SVRs with Gaussian kernel functions and Gaussian process regressors (GPR) with rational quadratic kernel functions. Finally, the quality scores provided by the regressors trained on different deep features extracted with the help of different CNN architectures are fused together to obtain the perceptual quality of a given video sequence.

### 3.1. Frame-Level Feature Extraction

**Figure 2:**
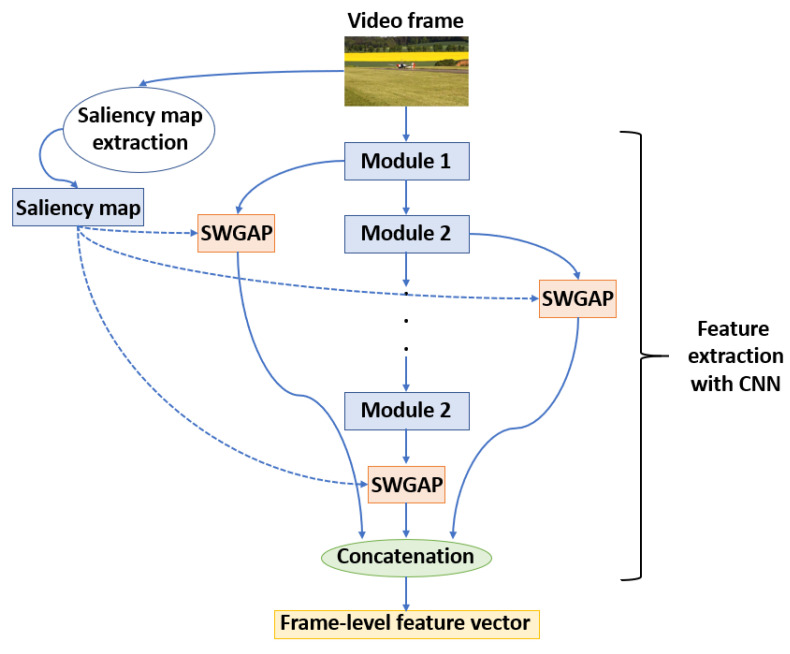
Illustration of frame-level feature extraction.

The workflow of the frame-level feature extraction is illustrated in Figure 2. As previously mentioned, a diverse set of pre-trained CNNs was applied to extract frame-level feature vectors independently from each other. Specifically, AlexNet [67], VGG16 [68], ResNet18 [69], ResNet50 [69], GoogLeNet [70], GoogLeNet-Places365 [70], and InceptionV3 [71] were considered for this purpose. Excluding GoogLeNet-Places365 [70], these architectures were pretrained on ImageNet [58] which contains more than one million images and 1000 semantic categories. On the other hand, GoogLeNet-Places365 [70] was trained on the Places-365 [59] database which consists of 18 million training images from 365 scene categories (i.e., art studio, beauty salon, biology laboratory, etc.). To extract frame-level features, saliency weighted global average pooling (SWGAP) layers—which is the contribution of this study—are attached to certain modules of the base models. As pointed out in previous works [72,73,74,75], considering multiple level of deep features is able to improve perceptual quality estimation, as CNNs capture image features at multiple levels. Table 2 summarizes the considered modules of the applied pre-trained CNNs and the length of the extracted feature vectors. Specifically, it can be seen that the features of the convolutional modules were used in case of AlexNet [67], VGG16 [68], while the features of the residual and Inception modules were utilized in case of ResNet18 [69], ResNet50 [69] and GoogLeNet [70], GoogLeNet-Places365 [70], InceptionV3 [71], respectively.

**Figure 3:**
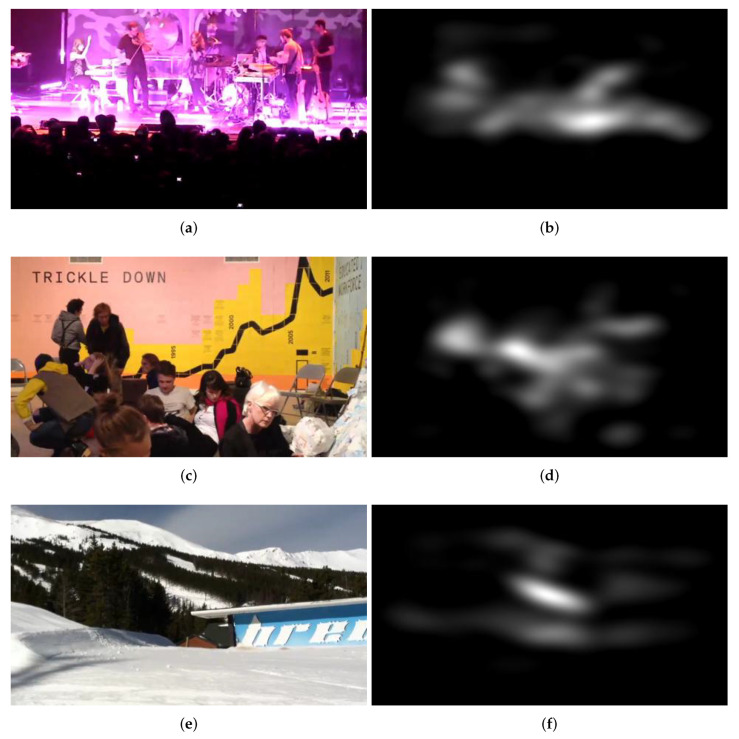

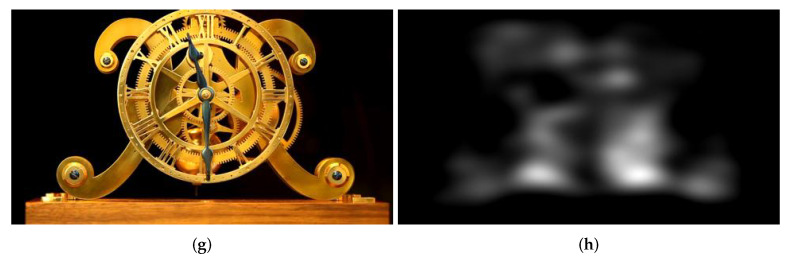
Illustration of saliency map extraction: (**a**,**c**,**e**,**g**) input video frames and (**b**,**d**,**f**,**h**) saliency maps of the input video frames obtained by the method of Li et al. [79].

Global average pooling (GAP) layers are usually used in CNNs to enforce correspondence between feature maps and the number of semantic categories and by this to enable the training of networks on images with various resolution [76]. Another common application of GAP is extracting resolution independent visual features from images with the help of a CNN. In this paper, we improve GAP to SWGAP for feature extraction using visual saliency. Namely, visual saliency algorithms deal with finding the most outstanding parts of a digital image from a perceptual point of view [77]. From the perspective of perceptual quality estimation, it is also very essential that human beings tend to fixate on some particular regions of the image during the first three seconds of the observation [78]. Motivated by the above observation, SWGAP is proposed for feature extraction to emphasize those regions which are salient to the human visual system. Namely, SWGAP performs a weighted arithmetic operation between an F(·,·) feature map of a CNN and the resized (bilinear interpolation is applied) S(·,·) saliency map of the input image. Formally, it can be written as

(1) $$\sigma =\frac{\sum_{i=1}^{M}\sum_{j=1}^{N}S\left(i,j\right)\cdot F\left(i,j\right)}{\sum_{i=1}^{M}\sum_{j=1}^{N}S\left(i,j\right)},$$

where σ denotes the output value of SWGAP for one feature map. Further, *M* and *N* stand for the height and the width of the feature map, respectively. The coordinates of the feature maps and the resized saliency map are denoted by *i* and *j*. In this study, the method of Li et al. [79] was applied to determine the saliency map of a video frame due to its low computational costs. Figure 3 depicts several video frames and their saliency maps.

### 3.2. Video-Level Feature Extraction

As previously mentioned, the frame-level feature vectors obtained with the help of a CNN architecture are temporally pooled together to compile one feature vector that characterizes the whole video sequence. In this study, the average pooling of frame-level feature vectors were utilized to this end. Formally, the following can be written:

(2) $$V_{i}^{\left(k\right)}=\frac{1}{N}\sum_{j=1}^{N}F_{i}^{j},$$

where *N* is the number of frames found in the given video, $F_{i}^{j}$ stands for the *i*th entry of the *j*th frame-level feature vector, while $V_{i}^{\left(k\right)}$ denotes the *i*th entry of the feature vector that characterizes the whole video sequence obtained by the *k*th CNN architecture. The $V^{\left(k\right)}$ feature vectors are mapped onto perceptual quality scores independently from each other by machine learning techniques. Specifically, we made experiments with two different regression techniques, such as SVRs with Gaussian kernel functions and GPRs with rational quadratic kernel functions. To obtain the estimated perceptual quality of a video sequence, the arithmetic mean or the median of the regressors’ outputs is taken.

## 4. Materials

In this section, the applied benchmark VQA databases (Section 4.1) and the applied evaluation protocol (Section 4.2) are described.

### 4.1. Applied Benchmark VQA Databases

In this paper, two large authentic VQA databases—KoNViD-1k [7] and LIVE VQC [26]—are used to evaluate the proposed method and other state-of-the-art algorithms. The videos of KoNViD-1k [7] were collected from the YFCC100m [80] database and evaluated in a large-scale crowd-sourcing experiment [81] involving 642 human observers who generated at least 50 quality ratings per video. The videos’ resolution is 960×540 and the MOS ranges from 1 to 5.

In [26], Sinno and Bovik compiled a VQA database containing 585 unique video sequences with authentic distortions captured by 80 different users with 101 different camera devices. Similarly to KoNViD-1k [7], the videos were evaluated in a large-scale crowd-sourcing experiment [82] involving 4776 human observers who produced more than 205,000 quality ratings. In contrast to KoNViD-1k [7], it contains videos with various image resolutions and the MOS ranges from 0 to 100. Unlike KoNViD-1k [7], LIVE VQC [26] has no fixed image resolution.

**Figure 4:**
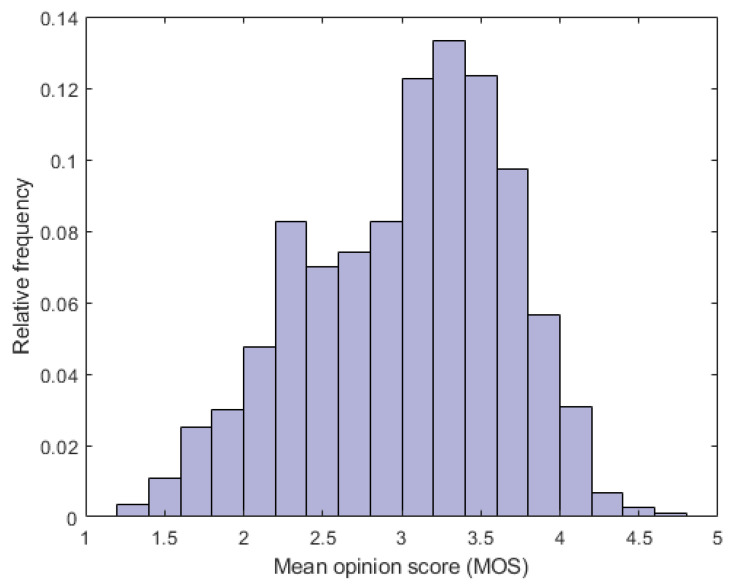
Empirical distribution of MOS in KoNViD-1k [7].

**Figure 5:**
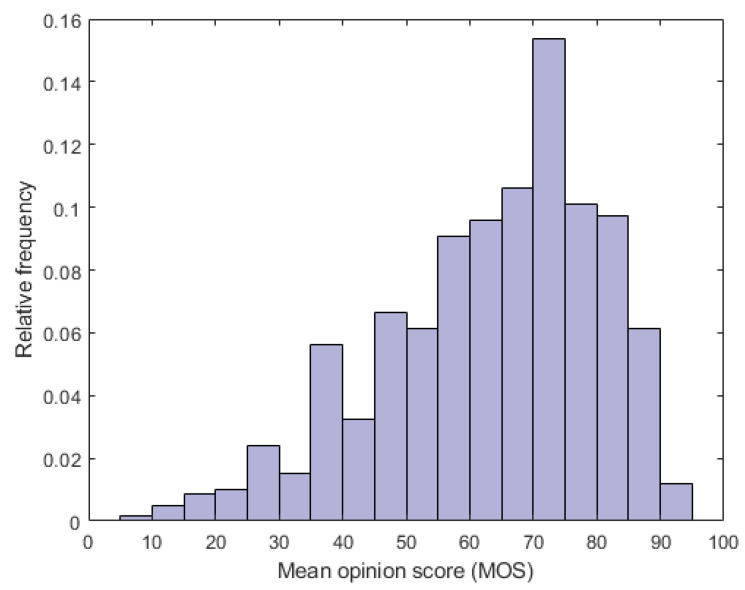
Empirical distribution of MOS in LIVE VQC [26].

**Figure 6:**
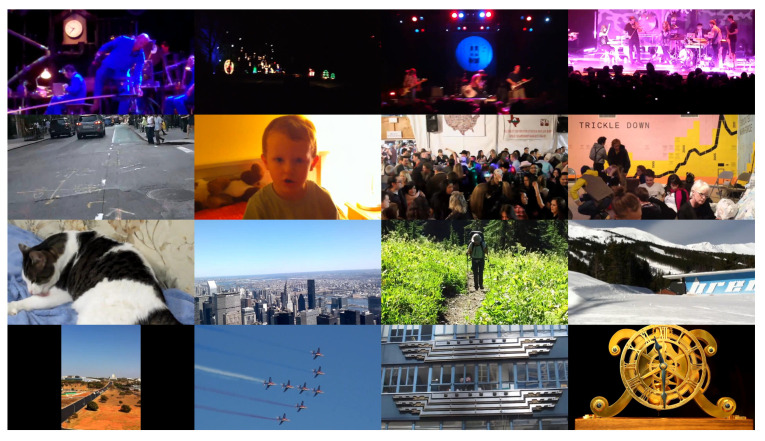
Illustration of several videos from KoNViD-1k [7] VQA benchmark database.

**Figure 7:**
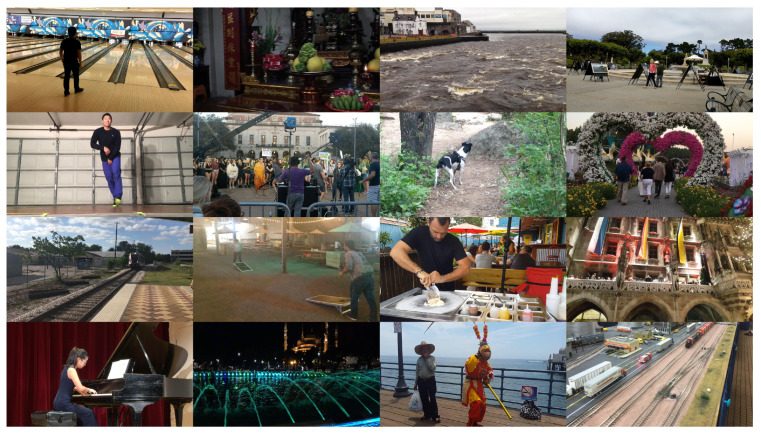
Illustration of several videos from LIVE VQC [26] VQA benchmark database.

The fundamental properties of the utilized VQA databases are summarized in Table 3. Further, the MOS distributions found in KoNViD-1k [7] and LIVE VQC [26] are depicted in Figure 4 and Figure 5, respectively. Figure 6 illustrates several videos from KoNViD-1k [7] VQA benchmark database. Similarly, Figure 7 depicts several videos from LIVE VQC [26].

### 4.2. Evaluation Protocol

The evaluation of VQA algorithms is based on determining the correlation between the ground-truth scores of a VQA database and the predicted scores given by the algorithm. In the literature, Pearson’s linear correlation coefficient (PLCC) and Spearman’s rank order correlation (SROCC) are applied. As already mentioned, KoNViD-1k [7] and LIVE VQC [26] are used to assess the proposed and other state-of-the-art methods. To this end, a VQA database is randomly divided into a training set (~80% of videos) and a test set (~20% of videos) to train a VQA method. This process is repeated 1000 times. Further, median PLCC and SROCC are reported in this paper. As suggested by Sheikh et al. [83], a non-linear mapping between the predicted and the ground-truth scores is executed before the calculation of PLCC. Specifically, a logistic function with five parameters is used to this end. Formally, this logistic function can be given as

(3) $$Q=\beta_{1}\left(\frac{1}{2}-\frac{1}{1+e^{\beta_{2}\left(Q_{p}-\beta_{3}\right)}}\right)+\beta_{4}Q_{p}+\beta_{5},$$

where $\beta_{i},i=1,...,5$ stand for the fitting parameters. Between datasets *A* and *B* with element number of *m*, PLCC is computed as

(4) $$PLCC\left(A,B\right)=\frac{\sum_{i=1}^{m}\left(A_{i}-\bar{A}\right)\left(B_{i}-\bar{B}\right)}{\sqrt{\sum_{i=1}^{m}{\left(A_{i}-\bar{A}\right)}^{2}}\sqrt{\sum_{i=1}^{m}{\left(B_{i}-\bar{B}\right)}^{2}}},$$

while SROCC is determined as

(5) $$SROCC\left(A,B\right)=\frac{\sum_{i=1}^{m}\left(A_{i}-A^{\prime }\right)\left(B_{i}-B^{\prime }\right)}{\sqrt{\sum_{i=1}^{m}{\left(B_{i}-B^{\prime }\right)}^{2}}\sqrt{\sum_{i=1}^{m}{\left(B_{i}-B^{\prime }\right)}^{2}}},$$

where $\bar{A}$ and $\bar{B}$ are the averages of set *A* and *B*, respectively. Moreover, $A^{\prime }$ and $B^{\prime }$ stand for the middle ranks of set *A* and *B*, respectively.

The proposed algorithm was implemented in MATLAB R2021a using the functions of the Image Processing, Machine Learning and Statistics, Deep Learning, and Parallel Computing Toolboxes. The details about the applied computer configuration is summarized in Table 4.

## 5. Results

In this section, the experimental results and analysis are presented using the benchmark databases and evaluation protocol described in Section 4. Section 5.1 summarizes the results of an ablation study so the design choices in the proposed method can be justified. In Section 5.2, a performance comparison in terms of PLCC and SROCC is presented against other state-of-the-art NR-VQA algorithms.

### 5.1. Ablation Study

In this subsection, an ablation study is presented to reason the design choices of the proposed method with respect to different feature extraction and regression techniques. Moreover, we demonstrate that the decision fusion of multiple deep architectures significantly improves the performance of NR-VQA. To this end, KoNViD-1k [7] database was applied in this ablation study using the evaluation protocol summarized in Section 4.2. The results are summed up in Table 5, Table 6, Table 7 and Table 8. From these results, it can be clearly seen that GPRs with rational quadratic kernel function provides much higher performance than SVRs with Gaussian kernel function in all possible cases. With regard to the decision fusion method, we can observe that the simple average is more favorable to the estimation performance than taking the median of the regressors’ outputs. More importantly, it can be clearly observed that the fusion of multiple CNNs’ results improves the prediction performance by a large margin. Further, the substitution of GAP layers with the proposed SWGAP layers is also able to improve the performance, as SWGAP applies visual saliency weighted average instead of simple arithmetic mean and by this those image regions can be emphasized which are salient to the HVS. As a result, GPRs with rational quadratic kernel functions, SWGAP layers, and arithmetic average as decision fusion were applied in the proposed method which is code-named as *SWDF-DF-VQA* in the followings.

### 5.2. Comparison to the State-of-the-Art

To compare the introduced *SWDF-DF-VQA* method to the state-of-the-art, we gathered eight NR-VQA algorithms including NVIE [84], V.BLIINDS [36], VIIDEO [85], 3D-MSCN [86], ST-Gabor [86], 3D-MSCN+ST-Gabor [86], FDD-VQA [87], and FDD+Perceptual-VQA [87] whose source codes were released by their authors. Obviously, the above mentioned algorithms were evaluated exactly the same way as the proposed method which is described in Section 4.2. Further, the performance results of eleven other methods, such as FC Model [45], STFC Model [45], STS-SVR [88], STS-MLP [88], ChipQA [89], QSA-VQM [50], Agarla et al. [51], Jiang et al. [90], MLSP-VQA-FF [6], MLSP-VQA-RN [6], and MLSP-VQA-HYB [6], were copied from the corresponding papers to give a more comprehensive comparison to the state-of-the-art. The results are summarized in Table 9 and Table 10. From these results, it can be concluded that the proposed method is capable to outperform the state-of-the-art by a large margin. According to Table 9, the proposed SWDF-DF-VQA outperforms the second best performing QSA-VQM [50] and MLSP-VQA-FF [6] by 0.046 and 0.036 in terms of PLCC and SROCC, respectively. Table 10 reports similar observations. Namely, the proposed method exceeds the second best algorithm’s performance by 0.025 and 0.029 in terms of PLCC and SROCC. As a result, a new state-of-the-art was set to authentic distortions.

## 6. Conclusions

In this paper, we presented a novel deep learning based approach for NR-VQA that utilizes a set of in parallel pre-trained CNNs for feature extraction. The main idea behind this layout was that a set of pre-trained CNNs can capture possible image distortions more versatitely than a single network. Specifically, temporally pooled and saliency weighted deep feature vectors were compiled with the help of multiple CNNs. Subsequently, these feature vectors were mapped onto perceptual quality scores and a decision fusion method was applied on them to obtain the quality rating of the whole video sequence. We demonstrated with extensive experimental results that such a arrangement of deep feature extraction and decision is able to improve the prediction performance by a large margin compared to single, deep architectures. Further, the proposed method was compared to other modern NR-VQA algorithms on two large benchmark VQA databases containing authentic distortions. Extensive experiments proved that the proposed method sets a new state-of-the-art on authentic distortions. Considering the achieved results, there are several directions for future research. For example, it is worth studying to combine motion and deep features to better characterize video distortions. In addition, a feature-level fusion of CNNs can be also a beneficial direction to reduce training time and computational costs.

## Figures and Tables

**Table 1:** Applied pre-trained CNN architectures and some of their main characteristics.

Network	Depth	Size	Parameters (Millions)	Image Input Size
AlexNet [67]	8	227 MB	61.0	227×227
VGG16 [68]	16	515 MB	138.0	224×224
ResNet18 [69]	18	44 MB	11.7	224×224
ResNet50 [69]	50	96 MB	25.6	224×224
GoogLeNet [70]	22	27 MB	7.0	224×224
GoogLeNet-Places365 [59,70]	22	24.3 MB	6.4	224×224
InceptionV3 [71]	48	89 MB	23.9	299×299

**Table 2:** Summary about the applied CNNs. The applied modules in feature extraction and the length of the extracted frame-level feature vectors are given.

Base CNN	Module	Length of Feature Vector
AlexNet	convolutional	1376
VGG16	convolutional	4224
ResNet18	residual	1920
ResNet50	residual	15,104
GoogLeNet	Inception	5488
GoogLeNet-Places365	Inception	5488
InceptionV3	Inception	10,048

**Table 3:** Overview about the applied VQA databases.

Attribute	KoNViD-1k [7]	LIVE VQC [26]
#Videos	1200	585
#Devices	>164	101
#Test subjects	642	4776
Format	MP4	MP4
Distortion	authentic	authentic
Test environment	crowdsourcing	crowdsourcing
Resolution	960×540	320×240–1920×1080
Duration	8 s	10 s
Frame rate	23–29 fps	19–30 fps

**Table 4:** Computer configuration.

Computer model	STRIX Z270H Gaming
Operating system	Windows 10
CPU	Intel(R) Core(TM) i7-7700K CPU 4.20 GHz (8 cores)
Memory	15 GB
GPU	Nvidia GeForce GTX 1080

**Table 5:** Performance of different base architectures and decision fusion methods using GAP layers for feature extraction and SVRs with Gaussian kernel function for regression. Median PLCC and SROCC were measured over 1000 random train–test splits.

Base CNN	PLCC	SROCC
AlexNet	0.735	0.734
VGG16	0.736	0.735
ResNet18	0.739	0.738
ResNet50	0.757	0.755
t6 GoogLeNet	0.741	0.739
GoogLeNet-Places365	0.712	0.711
InceptionV3	0.763	0.760
All—median	0.829	0.828
All—average	0.833	0.832

**Table 6:** Performance of different base architectures and decision fusion methods using GAP layers for feature extraction and GPRs with rational quadratic kernel function for regression. Median PLCC and SROCC were measured over 1000 random train–test splits.

Base CNN	PLCC	SROCC
AlexNet	0.785	0.781
VGG16	0.786	0.782
ResNet18	0.788	0.787
ResNet50	0.789	0.789
GoogLeNet	0.790	0.790
GoogLeNet-Places365	0.772	0.770
InceptionV3	0.794	0.793
All—median	0.848	0.847
All—average	0.853	0.852

**Table 7:** Performance of different base architectures and decision fusion methods using SWGAP layers for feature extraction and SVRs with Gaussian kernel function for regression. Median PLCC and SROCC were measured over 1000 random train–test splits.

Base CNN	PLCC	SROCC
AlexNet	0.741	0.740
VGG16	0.739	0.740
ResNet18	0.743	0.742
ResNet50	0.763	0.760
GoogLeNet	0.746	0.744
GoogLeNet-Places365	0.718	0.716
InceptionV3	0.769	0.765
All—median	0.833	0.832
All—average	0.838	0.836

**Table 8:** Performance of different base architectures and decision fusion methods using SWGAP layers for feature extraction and GPRs with rational quadratic kernel function for regression. Median PLCC and SROCC were measured over 1000 random train–test splits.

Base CNN	PLCC	SROCC
AlexNet	0.789	0.788
VGG16	0.790	0.788
ResNet18	0.793	0.794
ResNet50	0.793	0.796
GoogLeNet	0.795	0.794
GoogLeNet-Places365	0.777	0.775
InceptionV3	0.800	0.798
All—median	0.852	0.850
All—average	0.856	0.856

**Table 9:** Comparison of *SWDF-DF-VQA* to the state-of-the-art on KoNViD-1k [7]. Median PLCC and SROCC values were measured over 1000 random train–test splits. The best results are in bold, while the second best results are underlined.

Method	PLCC	SROCC
NVIE [84]	0.404	0.333
V.BLIINDS [36]	0.661	0.694
VIIDEO [85]	0.301	0.299
3D-MSCN [86]	0.401	0.370
ST-Gabor [86]	0.639	0.628
3D-MSCN+ST-Gabor [86]	0.653	0.640
FDD-VQA [87]	0.654	0.640
FDD+Perceptual-VQA [87]	0.716	0.711
FC Model [45]	0.492	0.472
STFC Model [45]	0.639	0.606
STS-SVR [88]	0.680	0.673
STS-MLP [88]	0.407	0.420
ChipQA [89]	0.697	0.694
QSA-VQM [50]	0.810	0.810
Agarla et al. [51]	0.790	0.780
Jiang et al. [90]	0.788	0.789
MLSP-VQA-FF [6]	-	0.820
MLSP-VQA-RN [6]	-	0.780
MLSP-VQA-HYB [6]	-	0.790
SWDF-DF-VQA	**0.856**	**0.856**

**Table 10:** Comparison of *SWDF-DF-VQA* to the state-of-the-art on LIVE VQC [26]. Median PLCC and SROCC values were measured over 1000 random train–test splits. The best results are in bold, while the second best results are underlined. We denote by “-” when the data are not available.

Method	PLCC	SROCC
NVIE [84]	0.447	0.459
V.BLIINDS [36]	0.690	0.703
VIIDEO [85]	−0.006	−0.034
3D-MSCN [86]	0.502	0.510
ST-Gabor [86]	0.591	0.599
3D-MSCN+ST-Gabor [86]	0.675	0.677
FDD-VQA [87]	0.623	0.630
FDD+Perceptual-VQA [87]	0.694	0.705
FC Model [45]	-	-
STFC Model [45]	-	-
STS-SVR [88]	-	-
STS-MLP [88]	-	-
ChipQA [89]	0.669	0.697
QSA-VQM [50]	0.780	0.740
Agarla et al. [51]	0.780	0.740
Jiang et al. [90]	0.789	0.776
MLSP-VQA-FF [6]	-	0.720
MLSP-VQA-RN [6]	-	0.700
MLSP-VQA-HYB [6]	-	0.690
SWDF-DF-VQA	**0.814**	**0.805**

## Data Availability

The used datasets were obtained from publically open source datasets from: 1. KoNViD-1k: http://database.mmsp-kn.de/konvid-1k-database.html, (accessed on 6 March 2022); 2. LIVE VQC: https://live.ece.utexas.edu/research/LIVEVQC/index.html, (accessed on 6 March 2022).

## Abbreviations

CNN	convolutional neural network
DCT	discrete cosine transform
DF	decision fusion
FR-VQA	full-reference video quality assessment
GAP	global average pooling
GGD	generalized Gaussian distribution
GPR	Gaussian process regressor
HVS	human visual system
IPTV	internet protocol television
LIVE	Laboratory for Image and Video Engineering
MOS	mean opinion score
NR-VQA	no-reference video quality assessment
NSS	natural scene statistics
PDF	probability density function
QANV-PA	quality assessment for network video via primary analysis
RBF	radial basis function
RR-VQA	reduced-reference video quality assessment
SVR	support vector regressor
SWDF	saliency weighted deep features
SWGAP	saliency weighted global average pooling
VQA	video quality assessment
VQC	video quality challenge
